# Speeding Up the Implementation of Industry 4.0 with Management Tools: Empirical Investigations in Manufacturing Organizations

**DOI:** 10.3390/s20123469

**Published:** 2020-06-19

**Authors:** Rok Črešnar, Vojko Potočan, Zlatko Nedelko

**Affiliations:** University of Maribor, Faculty of Economics and Business, Razlagova ulica 14, 2000 Maribor, Slovenia; rok.cresnar@student.um.si (R.Č.); vojko.potocan@um.si (V.P.)

**Keywords:** industry 4.0, readiness, implementation, management tools, manufacturing organizations

## Abstract

The main purpose of this study is to examine how the use of management tools supports the readiness of manufacturing organizations for the implementation of Industry 4.0. The originality of the research is reflected in the exploration of the relationship between the use of the selected well-known management tools and their readiness for the implementation of Industry 4.0, which was assessed using a combination of two models—one developed by the National Academy of Science and Engineering (Acatech) and the other by the University of Warwick. The relationship was assessed by applying structural equation modeling techniques to a data set of 323 responses from employees in manufacturing organizations. The results show that the use of six sigma, total quality management, radio frequency identification, a balanced scorecard, rapid prototyping, customer segmentation, mission and vision statements, and digital transformation is positively associated with Industry 4.0 readiness. Inversely, outsourcing and strategic planning are negatively associated with Industry 4.0 readiness, while lean manufacturing, which is often emphasized as the cornerstone of Industry 4.0 implementation, is not associated with Industry 4.0 readiness in our study. These findings can help organizations to understand how to consider and measure readiness for the implementation of Industry 4.0 more comprehensively and present guidelines on how the use of management tools in manufacturing organizations can foster their implementation of Industry 4.0 principles.

## 1. Introduction

Globally, Industry 4.0 is coming to the forefront with its practices and is shaping the environment in which organizations work under the increased influence of more advanced digital technologies and business practices [1,2]. In general, Industry 4.0 can be thought of as a techno-organizational paradigm [3], where its fundamental goal relates to technological advances of organizational workings, but it is also changing other areas of organizations [1,4]. Although the ideas of Industry 4.0 are very popular and have been recognized as beneficial among industrial organizations [5,6], its practices have been hard to implement [4,7,8].

First and foremost, this is due to the fact that organizations are not yet ready to comprehensively integrate the technological solutions that Industry 4.0 is based upon [4,8]. Furthermore, the implementation of Industry 4.0 is also hindered by the complexities and necessary changes in organizational factors related to people, business model transformation, transformed strategic orientations, and organizational culture [9,10,11,12,13].

The challenges of implementing Industry 4.0 are particularly significant for management [14,15]. Forthcoming changes have a comprehensive impact on how organizations work and are therefore shifting the current understanding of the role of management [13,16,17]. Different factors related to management influence beneficial organizational workings in times of change [18]. Among them, the use of appropriate managerial concepts, practices, and solutions, commonly recognized as management tools, has significant implications for the successful workings of organizations [19,20]. The literature also offers evidence on the role of individual management tools as beneficial, or in some cases as a hindrance, in the context of the Industry 4.0 environment [21,22]. We can argue that the use of appropriate management tools is crucial for organizations’ success in dealing with changes [23,24] and organizations need to implement Industry 4.0 to achieve higher levels of organizational productivity and competitiveness in the market [1,25].

As organizations have increasingly been transforming their workings to be more in line with Industry 4.0, the response has been a brainstorm of fragmented ideas and arguments on which tools management can use to properly implement these principles with an absence of comprehensive empirical evidence. Here, the question of how ready organizations are to implement Industry 4.0 starts to become important, because the ability to implement these upgrades, and thus beneficially change organizational workings, can be indicative of an organization’s ability to compete in the market [26]. However, as has been recognized, to successfully operate any high-level organizational change, management needs a correct and comprehensive framework of tools that serve as a goal-oriented way of achieving the desired organizational workings [19,23]. Industry 4.0 is still in the initial stages of its development [1], but it has already had a positive effect on organizations in terms of productivity and beneficial workings [3,6,27]. The utilization of management tools through the years [28,29,30,31] has also had a positive effect. Therefore, it is important to consider and further examine the relationship between both paradigms (i.e., Industry 4.0 and management tools) in order to obtain more detailed cognitions that can be beneficial in terms of organizational workings for organizations that are implementing Industry 4.0. 

With the utilization of management tools, organizations can support and manage highly complex situations in a structured way [24]. On the one hand, for Industry 4.0 implementation, management tools that are digital or more recently developed seem to complement the endeavor best, such as six sigma [32], total quality management [33], radio frequency identification [34], digital transformation [35], etc. Besides, it is also critical to employ management tools with a long history of use in organizations, such as strategic planning. Strategic planning in particular enables the creation of a strategic roadmap that can serve as a plan on how to successfully implement Industry 4.0 in organizations [36]. On the other hand, some management tools that are frequently used, such as outsourcing, are understood not to have a beneficial influence on Industry 4.0 implementation [37]. For other tools, such as lean production and management, opinions among researchers are more biased about their effectiveness, because lean production can present a fundamental building block of Industry 4.0 [38,39], or an insufficient base to build on that cannot be upgraded [40,41]. Management tools’ role in Industry 4.0 implementation, however, is only suggested in the sense that the content of the specific tool can be aimed at helping to cope with the challenges of implementing and supporting Industry 4.0. However, this has not yet been proven empirically in a comprehensive model in which a plethora of management tools are simultaneously considered.

Two aspects are not well understood with regard to the above-outlined issues. Firstly, what it means to be ready to implement Industry 4.0 in a comprehensive manner, as it is evident that the concept is not well understood [42]. Various models used to measure readiness for Industry 4.0 have been presented, such as the commonly emphasized models developed by the National Academy of Science and Engineering (Acatech) [43] and the University of Warwick [44], as well as some other models [22,26,45,46,47]. However, they have not been tested to determine whether they can simulate and fit data with observations in terms of predicting the effect on other factors of organizational workings. Secondly, organizations do not have a sufficient understanding of which managerial tools can be used or avoided in synchronicity to successfully implement Industry 4.0, as the literature on these tools is fragmented. Furthermore, although the effectiveness of certain management tools is implied in the context of Industry 4.0, we do not know whether the application of these management tools in organizations is still good enough in tandem with Industry 4.0, in order to lead the organizations toward the successful implementation of Industry 4.0 principles.

In our comprehensive approach to the problem, we use the most extensively developed Industry 4.0 readiness models designed by Acatech [43] and the University of Warwick [44] to determine the level of readiness for the implementation of Industry 4.0. Then, we determine which commonly used management tools, as well as those associated with Industry 4.0 as aforementioned, can predict Industry 4.0 readiness for implementation, both in terms of their content and their empirical association. We comprehensively examine the considered research questions, with the goal of offering a reliable model, first to determine exactly what Industry 4.0 readiness consists of, and second to see which management tools can reliably predict the model.

This study offers valuable contributions to both theory and practice. Firstly, we have determined structurally reliable dimensions for measuring readiness for Industry 4.0 based on factor analysis and the main indicators of goodness of fit and reliability. Secondly, we provide the selection of management tools which predicts the readiness for Industry 4.0 implementation. Thirdly, we have tested the impact of the eleven most commonly used management tools, as well as those related to Industry 4.0, on dimesons for Industry 4.0 readiness, and determined which are important drivers and which are inhibitors when implementing Industry 4.0. These cognitions address the deficit in non-comprehensive descriptions on the right managerial approaches to employ in order to address the issue of Industry 4.0 implementation, which have, up until this point, only partially been presented and addressed in the fragmented literature.

## 2. Theoretical Background and Hypotheses Development

### 2.1. Digitalization and Industry 4.0

The phenomenon of digitalization has impacted society in a way that it can rival other significant events in the history of humans, such as the development of language and agriculture or the mastery of fire [48]. Digitalization is a physical process based on digital technologies, which can be defined as devices, gadgets, or protocols that are capable of gathering, representing, interpreting, etc. data from real-world situations and recently using artificial intelligence, they are capable of learning [1,48]. When further developed and adapted to industrial processes, digitalization forms a paradigm of Industry 4.0, where it is understood as a goal-oriented paradigm with the purpose of achieving digital and interconnected manufacturing systems [1]. More specifically, Industry 4.0 is a broader concept than digitalization; although predicated on it, it more comprehensively envelops the current paradigm of changing organizational workings to those that are more connected, open, customer-oriented, and flat in organizational structure, reflecting the trends in global markets [9]. The phenomenon of Industry 4.0 was introduced in 2011 in Germany, which is the country that has led the implementation of Industry 4.0 principles and developed its economy through the integration of innovative technology into manufacturing processes [14,26]. Despite its introduction having occurred several years ago, it is still difficult to unambiguously define Industry 4.0 [7,42], because there is no agreement on whether it presents the next stage in the industrial revolution, a transformation of the way in which organizations develop, or a systematic change in strategy for the development of society [14,49,50]. In other more operational terms, Industry 4.0 presents a joint systemic integration of internet technologies and the manufacturing environment, where advanced internet-based technologies govern manufacturing operations [51]. The term Industry 4.0, however, most likely presents the German political agenda to transform its economy with the implementation of advanced technology not only in organizations, but also in society [52]. Despite this, it is evident that there are certain clusters of technologies that have formed and now drive ongoing digital transformation endeavors, which may also offer the most appropriate base for Industry 4.0 consideration in our study [1,53]:

1. Internet of Things (IoT), which integrates various processes with information and communication technologies, cloud computing, smart objects, and machines, leading to cyber–physical systems [54,55,56];
2. Cyber–Physical Systems (CPS), which use advanced technologies, big data, and real-time two-way communications to control production (and broader business) processes and systems [57,58];
3. Smart Factories, which are smaller and decentralized production units that are digitalized and autonomous [26]. This stage of integration is resulting in processes being much more efficient and productive [59].

### 2.2. Industry 4.0 Readiness

In recent years, several models for measuring an organization’s readiness for Industry 4.0 have been introduced. The main idea behind is to assess the areas of organizational workings that Industry 4.0 will be changing and impacting and create a set of criteria to measure that change [26,43,45]. We define readiness for Industry 4.0 as a comprehensive indicator of the organizational ability to adopt and implement practices of Industry 4.0 both in the area of technology and soft aspects of organizational workings.

Some models are tailored for small and medium-sized enterprises because they tend to experience the most problems with Industry 4.0 implementation and focus on the definition of Industry 4.0 concepts while offering guidelines for strategy creation [22,46,47,60]. The next group of models is focused on organizational maturity as an indicator of the level of already established implemented principles [22,26,46,47,60,61]. Yet another group is focused on readiness, which implies the preparedness for the initial adoption or an upgrade of Industry 4.0 principles [44,51]. Finally, some of the models are focused on special areas of organizational workings, such as logistics [62,63] or readiness examined through lean manufacturing [39]. In this study, we consider readiness based on two models, which seems to be the most comprehensive approach and offers the most variety in terms of application with regards to the organizational size or cultural and economic context. In this study, we used two comprehensive models, one designed by the Acatech [43] and another designed by the University of Warwick [44], to consider various aspects of organizational workings that are supported in the literature as important areas of change. The two models are used to achieve the most comprehensive numbers of criteria through which we can assess the readiness for the implementation of Industry 4.0. Both models also correspond well with perceived managerial challenges of Industry 4.0 implementation [14] and address key issues in terms of Industry 4.0 implementation [15].

The two models considered encompass the following dimensions: (1) Products and services that will change in their development and digital attributes [64]; (2) concepts and technologies that will focus on integrating the pillars of Industry 4.0 to transform the manufacturing and communication processes [1]; (3) strategic and organizational features that will require focused endeavors to align business and processes with Industry 4.0 initiatives [49]; (4) a supply chain that will have to enable real-time communication and control over the inventory, as well as manage constant changes in the business environment [1,31]. Furthermore, it should adopt practices of rapid prototyping, such as 3D printing [65]; (5) business model transformation that will focus on the customer rather than its own processes [9,55] and should aim to become more sustainable [66]; (6) legal and policy aspects that will require all the procedures to be compliant with current requirements, such as general data protection regulation (also known as GDPR) [67]; (7) culture and openness that will have to reflect new trends of encouraging innovativeness, cooperation, openness, and creativity among employees [35]; and (8) an organizational structure that will have to be more agile, flatter, and decentralized with regards to the decision-making processes [1].

### 2.3. Management Tools

In terms of understanding an organization as a complex economic system that is constantly evolving [68], many different concepts, ideas, and practices have been developed which can serve as a tool that supports comprehensive organizational goals [19,69]. Following the development of management through six distinct phases of its evolution (i.e., classical, humanistic, systems, contingency, post-modernistic, and scientific values) [70], management ideas have also gained different content and with that, several options for their definitions. Namely, as [19] outline,

- Concept: A comprehensively developed framework and base for the consideration of a select idea;
- Methodology: A plethora of comparable and related methods, rules, and interdisciplinary postulates;
- Methods: Procedures focused on goals and specific problems employing useful, regular, and systemic approaches for setting and achieving goals;
- Techniques: Focusing on ways of handling technical aspects.

Tools can be understood as various concepts and have different epistemological meanings. We can define them, with the help of Webster’s Dictionary [71], as means that can help to perform certain operations. Examining the concept more deeply, authors have also proposed that management tools are a plethora of analytical instruments used by managers to help them when they are implementing management concepts [72,73]. Furthermore, they can support managers’ work at all levels of organizations, from the conception of an idea to its implementation and realization in practice, with the goal of supporting organizational workings [19,29].

With their constant use and success in supporting managers, two broader distinct groups of management tools became evident, reflecting the historical development of management as a function [19]. The first group of management tools is more traditional, reflecting early developed and long-standing managerial practices, such as outsourcing, mission and vision statements, customer segmentation, strategic planning, etc. [24,69]. The second group of management tools is more contemporary, formed with tools such as Radio Frequency Identification (RFID), lean manufacturing, rapid prototyping, or six sigma [74]. The origin of these contemporary management tools is two-fold: They were either needed for the support of information technology-related aspects, or they were developed to complement and enhance their predecessors, more traditional management tools [29]. Additionally, they reflect the current needs of organizational development for more digitalized organizations.

Booth groups of tools, based on their content and partial empirical research, are useful for supporting the processes of organizational transformation towards Industry 4.0 [22,75]. However, to enhance the cognitions and obtain a deeper insight into the impact that each of the below-outlined management tools has on the readiness for Industry 4.0, the connection between the usage of selected management tools and Industry 4.0 readiness should be more carefully examined. 

### 2.4. Key management Tools That Can Support Industry 4.0 Implementation

When considering that Industry 4.0 is first and foremost understood as a technological advancement of organizational workings [3,5], in research, scientists have directly proposed that the use of certain contemporary management tools can serve as a precursor for the implementation of Industry 4.0, for instance, RFID [34,76], rapid prototyping [77], etc. More traditional management tools have also been put forward as being beneficial to use in the environment of Industry 4.0 [20,78], for instance, customer segmentation [60], mission and vison statements [20], etc. Therefore, these and many more of the below-outlined tools, which are, on the one hand, the most commonly used and important influencers of organizational workings [19,24,31], and on the second hand, supported in the below-referenced literature to be in some way connected to Industry 4.0, are considered as key management tools in our study. However, as traditional management tools are used for a longer period and contemporary tools are considered to be a derivation of traditional management tools [19], we argue that they may complement each other and work beneficially in tandem [31]. Therefore, in terms of Industry 4.0, we argue that key contemporary management tools (six sigma, lean manufacturing, RFID, rapid prototyping, and total quality management) could be more at the forefront. However, traditional management tools (customer segmentation, a balanced scorecard, mission and vision statements, strategic planning, and outsourcing) should also have a prevalent role in governing strategic organizational transformation and the implementation of Industry 4.0.

However, drawing on the conclusion that the below-described multidisciplinary literature recognizes associations between the use of management tools that we consider to be key and Industry 4.0, we can postulate the following hypothesis:

**Hypothesis 1.**  *The use of key traditional and contemporary management tools is associated with the readiness for the implementation of Industry 4.0.*

The reason why this hypothesis is important to consider is due to the abundance of previous literature explaining the beneficial effect of the most commonly used management tools on organizational workings [22,24,31,72,73,79]. However, the changes that Industry 4.0 brings to organizations are huge [1,4,42] and thus far, we do not know whether the most commonly used management tools, and by extension the most popular and established managerial practices [29], are still an important factor in the organizational environment of Industry 4.0.

More precisely, we next describe a set of traditional and contemporary management tools that were selected for the study because researchers have proposed that the content of the specific tools has some connection to Industry 4.0 implementation, either fostering or hindering the processes. Both traditional and contemporary management tools can be beneficial for helping to implement organizational change in terms of Industry 4.0. However, because the nature of their origin and use is different, we present arguments for selected tools in said groups.

#### 2.4.1. Traditional Management Tools in the Context of Industry 4.0 Implementation

The traditional management tools selected for this study are among the most used tools and have proven to be beneficial for organizational workings [24,31,79,80]. Moreover, the individual traditional management tools employed for this study were selected based on their mentions in the literature regarding Industry 4.0 challenges. Therefore, we can postulate the following:

**Hypothesis 2.**  *The use of key traditional management tools has a significant impact on the readiness for the implementation of Industry 4.0*.

We consider the following tools. The first is outsourcing, which is a tool with the aim of relocating organizational activities outside or offshore, primarily due to cost reduction [81]. This may become less prominent because the integration of new technologies has made organizations more connected and efficient internally, so the need to move manufacturing or outsource parts of operations is not as popular among organizations [37,82].

**H2a.**  *The use of outsourcing is negatively associated with the readiness for the implementation of Industry 4.0*.

The second tool is strategic planning, which enables organizations to design and execute long-term goals [24]. For Industry 4.0, it can be essential, since it enables the creation of a strategic roadmap that serves as a plan on how to successfully implement Industry 4.0. Although each organization should focus on its own workings, because there is no common strategy for all organizations [36,58], strategic planning seems to have a positive contribution to Industry 4.0 implementation, as it provides a key starting point on a journey toward Industry 4.0 adoption.

**H2b.**  *The use of strategic planning is positively associated with the readiness for the implementation of Industry 4.0*.

The third tool is mission and vision statements, which enable organizations to create and comprehensively understand the strategies of organizational workings and development [24]. They are, similar to strategic planning, fundamental for creating and understanding a comprehensive organizational strategy towards Industry 4.0 implementation [60]. It is important that all of the organizational participants understand and reflect the organizational values, which helps to create an organizational culture [24,78,79,80,83,84] that is of fundamental importance in the Industry 4.0 environment [35].

**H2c.**  *The use of mission and vision statements is positively associated with the readiness for the implementation of Industry 4.0.*

The fourth tool helps to create customer clusters, with the purpose of addressing their needs individually [24], which is becoming necessary in society, where tailor-made services and products are coming to the forefront. Customer segmentation is so fundamental for Industry 4.0 that it is proposed as a criterion in certain readiness models [60]. Furthermore, Industry 4.0 business models are focused more on customers [9,66]. However, there are some concerns about whether Industry 4.0 is too focused on processes and not focused enough on customers [4].

**H2d.**  *The use of customer segmentation is positively associated with the readiness for the implementation of Industry 4.0.*

A balanced scorecard comprehensively supports organizational systems, including strategic management, change management, sustainability, etc. [85]. Because it also comprehensively supports information systems, it may have significant implications in determining how organizations implement changes [86] and also provides a useful base for determining manufacturing organizations’ performance indicators [87]. A balanced scorecard also enables the assessment of multiple criteria, which are relevant for Industry 4.0 implementation, from various standpoints, such as processes [85,86,87,88].

**H2e.**  *The use of a balanced scorecard is positively associated with the readiness for the implementation of Industry 4.0*.

#### 2.4.2. Contemporary Management Tools in the Context of Industry 4.0 Implementation

Industry 4.0 is, at its core, a technological advancement-based paradigm [1,8]. Accordingly, among contemporary management tools, we included tools that are based on the usage of information technology or tools, where their usage must be supported with information technology in order to capture the benefits of their usage [20]. We can postulate the following:

**Hypothesis 3.**  *The use of key contemporary management tools has a significant impact on the readiness for the implementation of Industry 4.0*.

Lean manufacturing and Industry 4.0 aim to increase productivity based on the advancement in technologies and seem to be complementary to one another, where lean production is considered the base for implementing Industry 4.0 [89]. There are also some similarities in the content, and arguments that the fundamental lean principle can be complementary to Industry 4.0 have been made [39]. More fundamentally, however, the ideas are very different; whilst lean manufacturing focuses on reducing complexity around processes, Industry 4.0 more comprehensively reduces complexity around people [90]. Therefore, lean manufacturing is already reaching its limits and, with the introduced complexities of Industry 4.0, its effectiveness can be limited [41,91].

**H3a.**  *The use of lean manufacturing has reached its limits; thus, it is negatively associated with the readiness for the implementation of Industry 4.0*.

Digital transformation is sometimes used as a synonym for Industry 4.0 [43], showing the fundamental aims that it had from the beginning. However, when considered as a management tool, these practices aim to integrate digital technologies into organizations’ strategic and operational processes. These practices were developed very recently in order to comprehensively address the issues related to the complexity of managing technological advancements [35,78]. However, it has also been pointed out that digital transformation, although beneficial for productivity [92], has not yet been effectively evaluated in terms of its effect and impact on social systems [48].

**H3b.**  *The use of digital transformation is positively associated with the readiness for the implementation of Industry 4.0.*

Rapid prototyping is a tool that first and foremost supports product development with software-based technologies, eliminating the need for physical prototypes. Furthermore, it can also be used to develop IoT [77]. Additionally, smart technologies of Industry 4.0 that are proposed are integrated with technologies that enable rapid prototyping [4], such as 3D printing [93], which has become an important competitive advantage, reducing the time from development to market.

**H3c.**  *The use of rapid prototyping is positively associated with the readiness for the implementation of Industry 4.0*.

Six sigma is a tool that aims to secure the continuous improvement of production systems with methods of process standardization and a reduction in the process variability [94]. Now, however, its new conceptual variant, Lean six sigma, is more enhanced and integrated with the concepts of Industry 4.0, such as big data [95]. Moreover, augmenting the concept enables the production processes to be comprehensively modeled [32]. Six sigma will, therefore, foster the implementation of Industry 4.0, as it strives to reduce the number of failures to a minimum.

**H3d.**  *The use of six sigma is positively associated with the readiness for the implementation of Industry 4.0*.

Total quality management (TQM) is a managerial concept that enables the consistency of organizational workings and its continuous improvement [96]. In today’s organizational environment, TQM is an enabler of a more comprehensive goal to achieve organizational excellence. Along the path toward excellence, organizations rely on principles of total quality management that are fundamental for excellence creation in Industry 4.0 [33,97] and is therefore an important building block for the implementation of Industry 4.0 principles.

**H3e.**  *The use of TQM is positively associated with the readiness for the implementation of Industry 4.0*.

Radio frequency identification (RFID) is sensor-based technology that tracks products and materials throughout the entire phase of production and warehousing [98]. In recent years, it has improved and it is very compatible with the industrial internet of things and CPS, which means that it is one of the systems enabling real-time communication between the machines [34]. RFID complements and reflects the main principles of Industry 4.0 by using information technology to comprehensively track products and materials, and provides a valuable database for Industry 4.0 operations in organizations [76].

**H3f.**  *The use of RFID is positively associated with the readiness for the implementation of Industry 4.0*.

## 3. Methods

### 3.1. Instrument

The instrument was, in the first part, focused on the demographic characteristics of respondents, namely, age, gender, education, position, and work experience. These demographic variables have been considered to be important controls in obtaining reliable results in previous managerial studies [19,31]. In the second part, we measured organizations’ readiness for the implementation of Industry 4.0. Questions were designed based on proposed areas of change in comprehensive models presented by Acatech [43] and the University of Warwick [44]. Questions were presented in groups encompassing statements about products and services, concepts and technologies, strategic and organizational features, the supply chain, business model transformation, legal and policy aspects, culture and openness, and the organizational structure. In the third part, we measured the use of key management tools. To determine which management tools should be considered with regards to the relevant research problem, we drew on cognitions from the multidisciplinary literature and studies on the use management tools that offered reliable frameworks [19,24,29,78].

### 3.2. Sample and Procedure

The participants for this study were obtained based on random sampling among Slovenian manufacturing organizations. We selected organizations using the Slovenian government repository of business information (i.e., AJPES). We used Statistical Classification of Economic Activities in the European Community (i.e., NACE) to select organizations for the study. Only organizations according to NACE C classification were selected for the study. NACE C classification comprises a manufacturing sector, where studies suggest that Industry 4.0 has the greatest effect [55,61,66,99,100,101]. A link to an online survey was sent to 2800 email addresses of employees with managerial positions, with a focus on top managers, found on company websites, or it was requested that the email was forwarded on to them. A survey was conducted between 19.11.2019 and 7.1.2020, and no more than two emails per organization were sent. The response rate was 7.96%, as we received 323 completed responses, which comprised a representative sample of the Slovenian manufacturing sector. This was also a large enough sample size to draw meaningful conclusions from the data analysis [102].

In the sample, there were 228 (70.6%) male respondents and 95 (29.4%) female respondents. Respondents were, on average, 42.98 years old, with a standard deviation of 10.63 years. With regard to the respondents’ position in the organization, there were 8.4% specialists, 2.5% lower managers, 12.1% middle managers, 22% top managers, 48.9% CEOs and owners, and 6.2% respondents with other roles and positions. On average, the respondents had 19.56 years of work experience, with a standard deviation of 10.64 years. In the sample, there were 22% micro-enterprises (1-9 employees), 39% small enterprises (10-49 employees), 26.6% medium-sized enterprises (50-249 employees), and 12.4% large enterprises (more than 250 employees).

### 3.3. Measures

**Industry 4.0 readiness:** To measure the dimensions of readiness for Industry 4.0, we used an 11-point interval scale. The respondents were able to select an answer between 0 (i.e., not implemented concept) to 10 (i.e., a fully implemented concept). The dimensions, and with them associated questions, were considered based on the proposed areas of change in the aforementioned comprehensive models for determining Industry 4.0 readiness [43,44]. Next, we conducted a principal component factor analysis with varimax rotation to extract the main dimensions of readiness based on 52 questions regarding various criteria that were considered based on the aforementioned comprehensive models. We extracted factors with factor score weights greater than 0.5 and with 72.2% of variance explained; Kaiser–Meyer–Olkin (KMO) = 0.922 (*p* < 0.001).

The extracted factors formed the following dimensions, which were used in further structural equation modeling (SEM) analysis as measured variables: manufacturing concepts, technologies, and technology-related strategic orientations (“MCTS”, N = 12; α = 0.952); leadership, employees, and innovation culture (“LEI”, N = 8; α = 0.917); information and communication technology, and process control (“ITPC”, N = 9; α = 0.912); organizational strategy and investments (“STRI”, N = 4; α = 0.879); business model (“BM”, N = 6; α = 0.849); customers and market orientation (“CM”, N = 4; α = 0.727); organizational structure and openness (“OSO”, N = 3; α = 0.802); and products and services (“PAS”, N = 3; α = 0.702). Those eight dimensions were further collapsed and combined to explain the factor of readiness for Industry 4.0 used in Table 1 (N = 8; α = 0.924). Furthermore, individual dimensions were also used in SEM to explain the latent construct of readiness for Industry 4.0.

**Management tool use:** To determine the frequency of management tool usage, the respondents were able to select an answer on a six-point interval-type scale ranging from one (i.e., never use the tool) to six (i.e., always use the tool). Similar scales were used in previous studies [19,29]. Individual management tools were later used in the SEM model as measurable variables.

### 3.4. Research Design

Our research incorporated the following steps:

Step 1: We used descriptive statistics to observe the state of the factor structure that explained readiness for Industry 4.0 implementation, collapsed into a single factor (N = 8; α = 0.924), and the usage of 11 considered management tools. We also outlined the impact of control variables, namely age, gender, position, and organizational size, on other variables of interest in the study. Mean values and standard deviations are presented for all outlined variables. Next, we examined the correlations between variables. 

Step 2: To test the postulated hypotheses, we utilized structural equation modeling techniques. Firstly, we examined a comprehensive factor structure of the proposed model and conducted a confirmatory factor analysis with AMOS 21 software using maximum likelihood estimation procedures. The model with one latent construct, composed of eight individual factors, which were calculated based on factor analysis (i.e., readiness for Industry 4.0) and eleven measurable variables (i.e., management tools), displayed an adequate reliability and fit with the hypothesized model and data (χ2 (N = 323, df = 72) = 222. 375, *p* < 0.001; GFI = 0.937; CFI = 0.960; RMSEA= 0.081; PCLOSE < 0.05 (.000) [103].

In testing for multicollinearity between management tool use and Industry 4.0 readiness, tolerance values ranged from 0.241 to 0.505 and VIF values ranged from 2.105 to 5.150, thus presenting no multicollinearity issues [104]. We used one instrument to measure independent and dependent variables. Therefore, there is a possibility of common method bias [105]. To further test for common method variance, we used exploratory factor analysis with no rotation and loaded all 63 items (i.e., 52 from Industry 4.0 readiness and 11 from management tool use) onto one single factor [105]. The new common latent factor explained 39.35% of the variance, which was below the threshold value of 50%, thus indicating no common method bias issues [106]. Furthermore, along with no multicollinearity issues, there were no correlations greater than 0.90, which could indicate the possible presence of common method bias [107].

## 4. Results

### 4.1. Descriptive Statistics

The means, standard deviations, and correlations among the studied variables are presented in Table 1.

The results in Table 1 reveal that traditional management tools are most often used which is reflecting the trends of the Western economies [69], i.e., strategic planning, followed by mission and vision statements and outsourcing. Next are more contemporary tools, including total quality management and digital transformation, reflecting their popularity in recent years [78]. Lean production, customer segmentation, a balanced scorecard, and rapid prototyping are not among the top tools used. Whilst six sigma and RFID are at the bottom in terms of usage, however, they can be considered among the top contemporary tools that support digitalization [32,34,94]. With regards to the construct of readiness for the implementation of Industry 4.0 ($\bar{y}$ = 6.38; σ = 1.18), it displays a high variability, as not all organizations are on the same level, but the mean value indicates relatively early stages of Industry 4.0 adoption among manufacturing organizations in the sample.

With regards to correlation analysis, the results reveal that the majority of management tools display significantly weak to moderate positive correlations between each other. This reflects the similarity between the content they possess [78]; the fact that management tools are often used together for the same goal [31]; or the synergetic effect resulting from various phases of managerial development [70], where, for instance, the content of a traditional management tool is reflected in a new contemporary management tool. 

In some cases, control variables display significant weak associations with the use of certain management tools and also explain the nature of organizational workings in our sample. For example, larger organizations have fewer female managers and less profound management roles than smaller organizations. The management level increases with the age of the respondent and the level of the management role is negatively associated with gender. With regards to the associations of control variables with management tool use, digital transformation, a balanced scorecard, RFID, six sigma, and strategic planning are more frequently used in larger organizations. Customer segmentation, lean production, and strategic planning are more frequently used among managers with higher positions. Finally, a balanced scorecard, mission and vision statements, and strategic planning are more frequently used among older managers.

The correlations between readiness for Industry 4.0 and management tools are all noteworthy and significant. Although their strength is weak to moderate, the connections are all positive. These results, coupled with a lack of empirically verified associations and the fragmented literature, allowed us to proceed with a more comprehensive examination of which management tools are associated with Industry 4.0.

### 4.2. Multivariate SEM Analysis

**Readiness for the implementation of Industry 4.0:** The results in Table 2 reveal that factors extracted from the confirmatory factor analysis significantly explain the latent construct of readiness for Industry 4.0, displaying positive SEM standardized regression weights above 0.730, and are significant at *p < 0.001* [108]. The model of the readiness for Industry 4.0 implementation can be considered reliable as the average variance extracted (AVE) is greater than 0.5, the composite reliability (CR) is above 0.7, and the Cronbach’s alpha is above 0.7 [109]. There is also no great variation among and within readiness factors.

**Figure 1:**
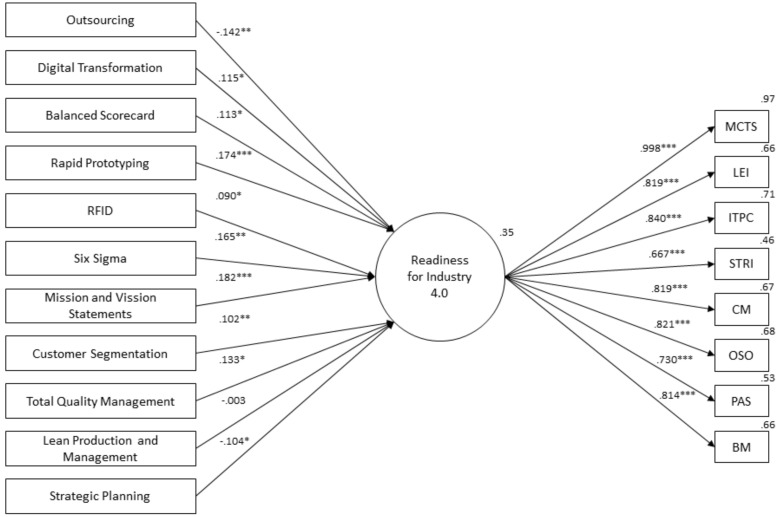
Structural equation model. Source: own elaboration; ***p < 0.001, **p < 0.05, and *p < 0.01; sample size is 323.

Obtaining a reliable factor structure for Industry 4.0 readiness allowed us to proceed with further analysis. Figure 1 shows the standardized regression weights and relationships among variables. In Table 3, the standardized direct effects (D) between readiness for Industry 4.0 and management tool use are presented. The standardized indirect effects (ID) are presented for the impact of management tool usage on factors comprising latent variable readiness for Industry 4.0.

First and foremost, the results in the structural equation model in Figure 1 show that the use of management tools explains 35% of the variance in readiness for Industry 4.0. All management tools considered in the analysis, except for lean production and management, are significantly associated with readiness for Industry 4.0 implementation. The most strongly positively related management tool to Industry 4.0 readiness is mission and vision statements (β = 0.182***), followed by rapid prototyping (β = 0.174***), six sigma (β = 0.165**), total quality management (β = 0.133*), digital transformation (β = 0.115*), a balanced scorecard (β = 0.113*), customer segmentation (β = 0.102**), and RFID (β = 0.090*). Management tools negatively and significantly associated with Industry 4.0 readiness are outsourcing (β = −0.142**) and strategic planning (β = −0.104**), while the association of lean production and management (β = −0.003) is negative, but insignificant.

Concerning the indirect effects (ID) listed in Table 3, it is evident that management tools that are positively associated with Industry 4.0 readiness in general, are also positively associated with the factors of readiness across the board. The same holds for negatively associated management tools. With regards to significant associations of indirect effects, customer segmentation, lean production, and outsourcing do not show any significant associations with individual readiness factors. Mission and vision statements, RFID, TQM, six sigma, rapid prototyping, a balanced scorecard, and digital transformation all show significant associations with individual readiness factors. Finally, strategic planning shows significant associations with CM, STRI, ITPC, and LEI readiness factors.

### 4.3. Hypotheses Analysis

Table 4 presents the results and outcomes with regards to hypotheses analysis. Established and long-used managerial concepts, techniques, methods, and methodologies still seem to be impactful under the new organizational environment that Industry 4.0 has created. Specifically, it is very important that, with regards to the readiness for Industry 4.0 implementation, the use of management tools explains 35% of the variance. The use of selected managerial tools thus has an important role in predicting the readiness for the implementation of Industry 4.0, confirmed by the reliable empirical model, and a significant portion of the explained variance [104,110] enables us to confirm Hypothesis 1.

Based on the obtained direct effect results presented in Table 3 and Figure 1 and supported by the indirect effect results shown in Table 3, we can conclude the following. With regard to the role of specific traditional management tools, we can confirm the following sub-hypotheses: *H2a, H2c, H2d,* and *H2e.* Additionally, we can reject the hypothesis *H2b*, as strategic planning exhibited a negative relationship with Industry 4.0 readiness.

In general, the use of traditional management tools displayed, in all cases, a significant association with Industry 4.0 readiness, thus confirming Hypothesis 2. 

In terms of more contemporary management tools, we can confirm the hypotheses *H3b, H3c, H3d, H3e,* and *H3f*. We can reject *H3a*, because lean manufacturing did not show any significant associations with Industry 4.0 readiness. The majority of contemporary management tools, except for lean manufacturing, exhibited significant associations with Industry 4.0 readiness, thus partially confirming Hypothesis 3.

## 5. Discussion

The results reflect the prior findings that put organizations in the initial or early stages of Industry 4.0 adoption [4,83,111]. Comparing our results with those from the University of Warwick model [44], we can observe that they paint a similar picture. Although measured on different scales, the considered factors of Industry 4.0 readiness show that, in general, organizations are positioned in the middle of the scale with regards to mean values. According to the results in Table 2, respectively all readiness factors’ mean values correspond with those obtained by the researchers from Warwick [44]. In terms of the levels of readiness proposed by Acatech [43], this would indicate that organizations are currently at stage three out of six, corresponding to the “visibility” stage, where changes in organizational philosophies occur and, for instance, data analysis is comprehensively integrated, rather than conducted individually. Based on these observations, it becomes evident that the relatviely low level of readiness in the case of Slovenia can become a significant issue as the need to implement Industry 4.0 in Slovenian organizations is especially important due to the great supply ties to German economy [31,112,113]. Taking this into account, using a plethora of appropriate management tools that can support Industry 4.0 implementation, should be the first step organizations should take in the near future [20].

Turning the attention to the use of management tools, the results also confirm that traditional management tools are still more frequently used, compared to others in our study. Thus confirming their effectiveness [24,31,74] and their benefits for organizational workings and success [19,78]. On the contrary, more contemporary management tools meant for supporting technological advances of organizational workings are not as frequently used. These results are in line with those obtained by previous studies [19,31], but their effectiveness for the implementation of Industry 4.0 is significant in our study and theoretically predicted in the literature. It is therefore evident that the tools which can support Industry 4.0 are not yet at the forefront with regard to their use. These findings support the above argumentations that organizations may currently be somewhere in the “midway” toward the implementation of Industry 4.0. 

With regard to the identified direct effects, traditional management tools, i.e., mission and vision statements, a balanced scorecard, and customer segmentation, have positive implications for Industry 4.0 readiness. This is expected because, for instance, mission and vision statements are one of the building blocks of organizational workings and a guide for the future [24]. In that sense, mission and vision statements support the organizational transformation [31,114], which is also true in the context of Industry 4.0 implementation [43]. Outsourcing is negatively associated with Industry 4.0 readiness, reflecting previous studies predicting that, due to the mediating impact of technologies that make internal processes more connected and efficient, outsourcing and similar practices are less needed [82], although the effects of “back-shoring” should be further examined in the context of Industry 4.0 [115]. The negative effect of outsourcing on Industry 4.0 may also be a bit surprising because when organizations outsource and focus on their core activities and processes, they must establish links with partners in order to have an overview over the outsourced processes [81]. Currently, it seems that these linkages are not either very tight nor based on information technology support. Further, the negative effects of strategic planning are curious with regard to the readiness for Industry 4.0. One would expect that strategic planning will also be tightly associated with Industry 4.0, since mission and vision statements show the strongest support for Industry 4.0 readiness among all management tools. This contradiction can imply that organizations have implemented the “transition toward Industry 4.0” in their missions and visions, but they had not fully implemented their vision in strategies and realized it on all levels and areas in organizations. This again reflects that manufacturing organizations are in the initial phases of Industry 4.0 adoption [13,115], especially with regards to strategic orientations, which was coincidently also the lowest scored readiness dimension in the University of Warwick model results [44]. Furthermore, there could be a logical, method-based explanation for the negative results, as organizations that have already implemented the concepts do not further strategize for their implementation.

Looking at contemporary management tools in this study, they all have positive associations with Industry 4.0 readiness (see Figure 1 and Table 3). Because they are based on different technologies and can support primary Industry 4.0 issues [7,45], they tend to fall in line with Industry 4.0 principles and be thought of as appropriate solutions by organizations. We can argue that those tools will importantly contribute to the implementation of Industry 4.0 principles in organizations. Lean manufacturing, however, is an exception, as it has not been proven to be significant in readiness for Industry 4.0 implementation, although it has been considered as an important vehicle for Industry 4.0 implementation [39,40,41]. As observed, Slovenian organizations do not use lean manufacturing as often as other tools, which is signaling that they are more focused on the processes [29]. The focus on a more comprehensive outlook that also incorporates people in the transformation might explain the difference and the limits of lean manufacturing [40]. This also supports our notion about the initial stages of Industry 4.0 implementation in organizations.

Surprisingly, the indirect effects show some very interesting associations. Outsourcing and customer segmentation, which are both traditional management tools, only show a significant direct effect on the Industry 4.0 readiness construct and no significant indirect effects on underlying factors. Both of those tools are of strategic importance in their nature, reflecting large-picture organizational decisions [82,116], and thus may have more of a systemic impact, as they are focused more on a comprehensive picture and not on the individual areas of organizational workings that are reflected in the obtained Industry 4.0 readiness model. Strategic planning also does not have a comprehensive indirect impact on Industry 4.0 readiness factors. It has no significant associations with the business model, organizational structure and openness, products and services, and manufacturing concepts and technologies. This could indicate that, with regards to planning for Industry 4.0, organizations usually do not focus on individual areas, or, it may indicate that these factors are more important than the rest. Alternatively, this can also reflect a lack of the strategic focus when implementing Industry 4.0, which can result in the poor implementation of Industry 4.0 with regards to the organizational strategy, a poor definition of goals related to Industry 4.0, and a poor understanding of Industry 4.0 implementation among employees [44]. In turn, such an approach may lead to the averseness of employees toward Industry 4.0 adoption [117], as well as the magnification of the thread of losing jobs due to the Industry 4.0 implementation [48,118].

To further understand, what our results reveal about the meaning of Industry 4.0 and what is required for its implementation, we need to first examine a broader context in which the results apply. Considering, authors tend to emphasize the initial hype, stating that Industry 4.0 represents the next industrial revolution [46,49,119] we add the following thoughts. Through the lens of our results, Industry 4.0 is, as of right now, another technical upgrade that is hard to implement [4], with the addition of certain principles of more contemporary business philosophies, capitalizing on technologies introduced in the third industrial revolution [120]. This can be observed through the mean value of the readiness construct ($\bar{y}\;$= 6.38; σ = 1.18) and its predicting factors, which are respectively low and highly variable. From this, the low usage of key contemporary management tools [19,31], and the problems with technological implementation in general [1,4,7] we can speculate that this is far from worldwide revolutionary changes in the way that the economy operates, and it might take decades to evolve into industrial revolution [121].

### 5.1. Theoretical Implications

This study offers a reliable model based on empirical investigations that confirm theoretical relations between the use of key management tools and Industry 4.0 readiness, which was previously only partially discussed in different studies [34,39,99,122] and with limited empirical data, or associations were only theoretically presupposed [60]. Second, we have confirmed and comprehensively defined an appropriate structure of criteria that should be considered when measuring readiness for the implementation of Industry 4.0 from previously presented comprehensive models [43,44]. These were merged in one study, whereas single models [62,122] have a limited ability to comprehensively explain readiness by not considering all of the important aspects of Industry 4.0 adoption [14,15]. For the first time, it can be observed, how the proposed dimensions of Industry 4.0 readiness from the considered models are formed, when subjected to factor analysis and applied in SEM. This may have significant implications for constructing a universally applicable model in the future. Third, by considering all of the examined relationships, we established a baseline model that simultaneously explains how the use of management tools can predict the readiness for the implementation of Industry 4.0 and further demonstrates exactly which management tools can support and hinder the processes of Industry 4.0 implementation. With that, we can go beyond fragmented studies on the impact of single management tools on Industry 4.0 [77,83,123], or their theoretical considerations [60].

### 5.2. Practical Implications

Organizations must have detailed information on how to implement Industry 4.0 based on empirical research [42,45] to design the appropriate steps. Following the practical needs of organizations, our study helps to understand the current state of how to determine readiness for the implementation of Industry 4.0, and how the use of key management tools can help to foster better readiness for organizations. For instance, the use of outsourcing is not recommended [82], as we found it to be mutually exclusive with the implementation of Industry 4.0. However, outsourcing is very frequently used in manufacturing organizations in Central Europe [31], which may impede the implementation of Industry 4.0 principles in this corner of the world. This implies that managers should re-think this trade-off between Industry 4.0 implementation and outsourcing. By extension, if organizations use outsourcing, they do not focus on improving internal processes or their relationship with customers, which could be considered instrumental in securing successful operations in Industry 4.0 [6]. Moreover, lean manufacturing has no real implications for Industry 4.0 readiness. In this situation, we can recommend that organizations do not base their Industry 4.0 implementation strategy on an already established utilization of lean production, but reduce the complexity of organizational workings more comprehensively, which is to say not only in the area of business processes. Organizations should foster the use of six sigma, TQM, RFID, a balanced scorecard, rapid prototyping, customer segmentation, and digital transformation practices, while strategically planning to improve their readiness for the implementation of Industry 4.0. Slovenian organizations in particular show a rather low readiness for Industry 4.0, which is a bit surprising, as Slovenian organizations are tightly related to the German economy, most often as suppliers [19]. This requires that Slovenian organizations, and other organizations with a similar level of readiness follow up in their development to catch up with focal organizations, which are considered as the producers of final goods [124], in order to become compatible with the settings in focal organizations [31,112,113]. This lag can be attributed to the small organizational size, a general lack of interest in adopting Industry 4.0, a lack of funds, short term agreements, inoperability of systems, etc. The current negative contribution of strategic planning to the manufacturing concepts, technologies, and strategic orientations, require managers to focus on the realization of the postulated mission and vision-related activities in the implementation of Industry 4.0. This could help to foster the role of strategic planning, which is in its core a very similar tool as mission and vision statements [24], and should have a substantial role in Industry 4.0 implementation [43,44,58]. Therefore, managers need to prepare detailed plans with regard to Industry 4.0 implementation, as well as workshops that should be organized to train the employees. This reflects the current talks among practitioners, who are emphasizing that we are at the very beginning of Industry 4.0 implementation, where we integrate Industry 4.0 into mission and vision statements and strategic goals, while its implementation for lower-level goals is still not fully realized. Therefore, managers need to recognize this and ensure that the strategic goals will be realized at lower levels and implemented in organizational practice as well.

With regards to where the practitioners should focus their efforts when implementing Industry 4.0, it is obvious from our results that the main focus should be first on implementing technological solutions. Technological factors ITPC and MCTS have the lowest mean values, but are the most important predictors of readiness. Although issues when implementing Industry 4.0 technologies are noted [4,7], organizations should, in order to catch up, focus their endeavors to design strategies where the primary focus is on technological integration, as the readiness of other organizational factors is well beyond those. 

As some authors argue, what will drive the implementation and workings of Industry 4.0 is the ability of organizations and their employees to be creative and have appropriate solutions at their disposal [125,126,127,128,129]. Positive contributions of more contemporary management tools, such as rapid prototyping, can support employees in creativity and innovation, while positive contributions of more traditional management tools, such as mission and vision statements, can support organizations in setting up the said environment.

Practical implications also concern academia. A study from [130] has outlined the importance of digital education in the context of Industry 4.0. Therefore, academia can capitalize on these results, renew the curricula and teaching methods, and emphasize digital resources of the most current studies as, for instance, yearly reports on the use of management tools are mainly published online [20,78].

### 5.3. Limitations

To put the results of the study in context, several limitations may be helpful. Firstly, the management tools that were used in the study were selected based on their content and theoretical propositions, indicating those that may have something in common with Industry 4.0. There are many more management tools available, such as advanced analytics and complexity reduction practices [20], that may have some implications for Industry 4.0 readiness, implying that our listing of management tools can be expanded. Secondly, the self-assessment scale was used to determine the outcomes, where employees were assessing readiness for the implementation of Industry 4.0 and the use of management tools. Self-assessment may have some implications for the results [130]. Thirdly, Slovenian manufacturing organizations are often suppliers for larger Western focal organizations, so Slovenia’s specific position in larger supply chains and its gradual transition to the free market economy may not reflect a state in Western developed countries [31]. Only NACE C organizations were selected for the study. Service organizations may use management tools differently in comparison to manufacturing organizations [19].

### 5.4. Future Research Directions

First, to more accurately confirm the model of readiness for the implementation of Industry 4.0, many more aspects of organizational workings should be considered, which, due to the early stage of research endeavors, might not yet have been found [43,44]. Secondly, due to the Slovenian-specific context in the development of its economy [31], many more Western developed economies should be considered. Research should be conducted in other countries that have different political, social, and economic settings, in order to test whether these associations are generally usable. Thirdly, management tool use is changing and new tools are emerging [20]. Once their effectiveness is proven, they should also be examined for their connection with Industry 4.0 implementation. Fourthly, contrary to the hype, as a whole, we still do not understand whether the changes in existing technologies are justified in saying that the next industrial revolution will truly be based on the aforementioned technological upgrades. From the standpoint of organizational workings, organizational outputs should be monitored across the globe to obtain a better idea of the changes and to understand whether it is worth studying the phenomenon of Industry 4.0 respectively as the new industrial revolution. Fifth, our results with regards to the neutral impact of lean manufacturing on Industry 4.0 readiness also challenge existing studies, which have suggested that lean manufacturing is the cornerstone for Industry 4.0 adoption [90,91,122]. This needs to be further examined. It would also be interesting to examine, how much new technologies, implemented under Industry 4.0, will contribute to the sustainability of organizations [131] and which management tools can support sustainable development in the context of Industry 4.0. Furthermore, as certain management tools can support innovation [20], and Industry 4.0 is also based on innovation [125,126,127,128], it should be examined which management tools can support the organization’s creativity and innovative behavior of employees under the circumstances of Industry 4.0.

## 6. Conclusions

This study provides an insight into the relationships between the use of selected well-known management tools and the organization’s readiness for the implementation of Industry 4.0. We developed a reliable model for determining the readiness for the implementation of Industry 4.0 in manufacturing organizations, by combining two comprehensive Industry 4.0 readiness models. The developed model more precisely addresses the areas where changes occur in organizations in the context of Industry 4.0 implementation. Next, our study enhances the Industry 4.0 readiness model with the integration of commonly used management tools that can predict readiness for Industry 4.0. The main contribution of our study comes from the results, which outline key management tools, considered by their content to be appropriate to use when implementing Industry 4.0, and how is their use beneficial to these endeavors. Those tools are six sigma, TQM, RFID, a balanced scorecard, rapid prototyping, customer segmentation, and digital transformation practices. Others, such as outsourcing and strategic planning, may have negative implications for implementing Industry 4.0. Further, we outlined that lean manufacturing, as a respectively crucial tool for Industry 4.0, has no significant implications for Industry 4.0 readiness, suggesting that the practice may have reached its limits of effectiveness. This study indicates that several well-known management tools that have been used for a long period, as well as other contemporary management tools, can have a significant effect on beneficial organizational workings under the circumstances of Industry 4.0 implementation. Additionally, for the first time, we can observe from the factor analysis exactly how dimensions of readiness can be combined in a more comprehensive structure and more accurately describe where the phenomenon is impacting organizational workings. The model showed that dimensions of Industry 4.0 readiness go beyond technological advances and are significantly changing other areas of organizational workings, although it should be stated that technological factors have the most influence on Industry 4.0 readiness. This is the first step in determining a universal model for measuring readiness for the implementation of Industry 4.0. 

## Figures and Tables

**Table 1:** The means (M), standard deviations (SD), and correlations among the studied variables.

Variable	M	SD	1	2	3	4	5	6	7	8	9	10	11	12	13	14	15	16
1 Age	42.98	10.64	1															
2 Gender	1.29	0.46	−0.89	1														
3 Position	4.19	1.30	0.248 **	−0.117 *	1													
4 Organizational size	2.29	0.95	−0.021	−0.136 *	−0.114 *	1												
5 Outsourcing	3.89	0.77	0.084	−0.108	0.109 *	0.053	1											
6 Digital transformation	3.85	0.75	0.065	−0.081	0.008	0.122 *	0.370 **	1										
7 Balanced scorecard	3.70	0.86	0.117 *	−0.103	0.079	0.150 **	0.429 **	0.567 **	1									
8 Rapid prototyping	3.62	0.99	0.101	−0.088	0.066	0.030	0.281 **	0.346 **	0.505 **	1								
9 RFID	2.12	0.84	−0.001	−0.042	−0.021	0.118 *	0.288 **	0.308 **	0.447 **	0.320 **	1							
10 Six Sigma	2.81	0.95	0.032	−0.073	0.029	0.152 *	0.371 **	0.343 **	0.546 **	0.364 **	0.510 **	1						
11 Mission and vision statements	4.26	0.74	0.141 *	−0.046	0.050	0.088	0.471 **	0.461 **	0.484 **	0.275 **	0.308 **	0.293 **	1					
12 Customer segmentation	3.80	0.79	0.025	0.037	0.128 *	−0.103	0.286 **	0.110 *	0.266 **	0.212 **	0.143 *	0.060	0.274 **	1				
13 Total quality management	3.87	0.84	0.071	−0.093	0.108	0.064	0.562 **	0.361 **	0.545 **	0.440 **	0.358 **	0.603 **	0.465 **	0.264 **	1			
14 Lean production and lean management	3.80	0.87	0.057	−0.092	0.112 *	0.084	0.505 **	0.120 **	0.621 **	0.445 **	0.300 **	0.549 **	0.362 **	0.240 **	0.627 **	1		
15 Strategic planning	4.30	0.68	0.146 *	−0.040	0.118 *	0.169 **	0.490 **	0.367 **	0.510 **	0.364 **	0.216 **	0.341 **	0.591 **	0.325 **	0.465 **	0.446 **	1	
16 Readiness for Industry 4.0	6.38	1.18	0.100	−0.020	-0.005	0.108	0.218 **	0.412 **	0.507 **	0.420 **	0.386 **	0.393 **	0.362 **	0.216 **	0.383 **	0.373 **	0.298 **	0.298 **

Notes: ** *p* < 0.001 and * *p* < 0.05; sample size is 323.

**Table 2:** Confirmatory factor analysis with descriptive statistics for the Industry 4.0 readiness model in SEM.

Construct	Measure	Mean	Std. Deviation	Standardized Direct Item Loading SEM	CR	AVE	Cronbach’s Alpha
Readiness for Industry 4.0	STRI	6.39	1.54	0.667 ***			
	CM	7.74	1.17	0.819 ***			
	OSO	6.99	1.52	0.821 ***			
	PAS	6.28	1.44	0.730 ***	0.940	0.669	0.924
	MCTS	5.08	1.54	0.998 ***			
	LEI	7.34	1.38	0.819 ***			
	ITPC	5.08	1.52	0.840 ***			
	BM	6.12	1.41	0.814 ***			

Notes: *** *p* < 0.001; sample size is 323.

**Table 3:** Standardized direct and indirect effects among variables.

Variable	Strategic Planning	Lean Manufacturing	TQM	Balanced Scorecard	Outsourcing	Customer Segmentation	Mission and Vision Statements	Six Sigma	RFID	Rapid. Prototyping	Digital Transformation
Read. (D)	−0.104 *	−0.003	0.133 *	0.113 *	−0.142 **	0.102 **	0.182 ***	0.165 **	0.090 *	0.174 ***	0.115 *
BM (ID)	−0.087	−0.002	0.108 *	0.092 *	−0.116	0.083	0.148 *	0.134 *	0.073 **	0.141 **	0.094 **
PAS (ID)	−0.078	−0.002	0.097 *	0.083 *	−0.104	0.075	0.133 **	0.120 **	0.065 **	0.127 **	0.084 **
OSO (ID)	−0.087	−0.002	0.109 *	0.093 *	−0.117	0.084	0.149 **	0.135 **	0.074 **	0.143 **	0.094 **
CM (ID)	−0.087 *	−0.002	0.109 *	0.093 *	−0.117	0.084	0.149 ***	0.135 *	0.074 **	0.142 **	0.094 *
STRI (ID)	−0.072 *	−0.002	0.090 *	0.077 *	−0.096	0.069	0.123 **	0.112 *	0.061 **	0.118 *	0.078 *
ITPC (ID)	−0.089 *	−0.003	0.111 *	0.095 *	−0.120	0.086	0.153 **	0.138 *	0.075 **	0.146 **	0.097 **
LEI (ID)	−0.086 *	−0.002	0.108 *	0.092 *	−0.116	0.083	0.148 **	0.134 **	0.073 **	0.141 **	0.093 **
MCTS (ID)	−0.106	−0.003	0.133 *	0.113 *	-0.142	0.102	0.182 **	0.165 **	0.090 **	0.174 **	0.115 **

Notes: * *p* < 0.05, ** *p* < 0.01, and *** *p* < 0.001; sample size is 323.

**Table 4:** Hypotheses analysis.

Hypotheses	Sub-hypotheses	Relations	Effect	Result
Hypothesis 1	General	Management tools use→Industry 4.0 readiness	0.35% ***	Support
Hypothesis 2	*H2a*	Outsourcing→Industry 4.0 readiness	−0.142 **	Support
	*H2b*	Strategic planning→Industry 4.0 readiness	−0.104 *	Reject
	*H2c*	Mission and vision statements→Industry 4.0 readiness	0.182 ***	Support
	*H2d*	Customer segmentation→Industry 4.0 readiness	0.102 **	Support
	*H2e*	Balanced scorecard→Industry 4.0 readiness	0.113 *	Support
Hypothesis 3	*H3a*	Lean manufacturing and management→Industry 4.0 readiness	−0.003	Reject
	*H3b*	Digital transformation→Industry 4.0 readiness	0.115 *	Support
	*H3c*	Rapid prototyping →Industry 4.0 readiness	0.174 ***	Support
	*H3d*	Six Sigma →Industry 4.0 readiness	0.165 **	Support
	*H3e*	TQM→Industry 4.0 readiness	0.133 *	Support
	*H3f*	RFID→Industry 4.0 readiness	0.090 *	Support

Notes: * *p* < 0.05, ** *p* < 0.01, and *** *p* < 0.001; sample size is 323.
